# Long-period ocean-bottom motions in the source areas of large subduction earthquakes

**DOI:** 10.1038/srep16648

**Published:** 2015-11-30

**Authors:** Takeshi Nakamura, Hiroshi Takenaka, Taro Okamoto, Michihiro Ohori, Seiji Tsuboi

**Affiliations:** 1 Research and Development Center for Earthquake and Tsunami, Japan Agency for Marine–Earth Science and Technology, 3173-25 Showa-machi, Kanazawa-ku, Yokohama 236-0001, Japan; 2 Department of Earth Sciences, Okayama University, 3-1-1 Tsushima-Naka, Kita-ku, Okayama 700-8530, Japan; 3 Department of Earth and Planetary Sciences, Tokyo Institute of Technology, 2-12-1 Ookayama, Meguro-ku, Tokyo 152-8551, Japan; 4 Research Institute of Nuclear Engineering, University of Fukui, 1-2-4 Kanawa-cho, Tsuruga City, Fukui 914-0055, Japan; 5 Center for Earth Information Science and Technology, Japan Agency for Marine–Earth Science and Technology, 3173-25 Showa-machi, Kanazawa-ku, Yokohama 236-0001, Japan

## Abstract

Long-period ground motions in plain and basin areas on land can cause large-scale, severe damage to structures and buildings and have been widely investigated for disaster prevention and mitigation. However, such motions in ocean-bottom areas are poorly studied because of their relative insignificance in uninhabited areas and the lack of ocean-bottom strong-motion data. Here, we report on evidence for the development of long-period (10–20 s) motions using deep ocean-bottom data. The waveforms and spectrograms demonstrate prolonged and amplified motions that are inconsistent with attenuation patterns of ground motions on land. Simulated waveforms reproducing observed ocean-bottom data demonstrate substantial contributions of thick low-velocity sediment layers to development of these motions. This development, which could affect magnitude estimates and finite fault slip modelling because of its critical period ranges on their estimations, may be common in the source areas of subduction earthquakes where thick, low-velocity sediment layers are present.

Real-time seismic monitoring systems in deep ocean areas have been implemented in areas such as Canada^1^, Europe^2^, Japan^3^,^4^, Taiwan^5^, and the USA^6^, where suboceanic earthquakes frequently occur. Such observation systems allow rapid detection of signals from suboceanic earthquakes and develop azimuthal coverage of ocean areas, enhancing the precision of hypocentre determination and magnitude estimation of earthquakes. Analysis of ocean-bottom data has provided novel insights into oceanic seismic activity^7^, seismic structure^8^, background noise^9^, and acoustic wave propagation^3^.

In the Nankai trough area in southwestern Japan, where the Philippine Sea plate is subducting beneath the continental Amur plate, M8-class large subduction earthquakes have repeatedly occurred at intervals of 100–200 yr, including the 1944 Tonankai (*M*w 8.1) and the 1946 Nankai earthquakes (*M*w 8.1)^10^. In 2010, a permanent ocean-bottom observatory with 20 geophysical stations was deployed at water depths of 1900–4400 m near the trough area^4^. Each station is equipped with an acceleration seismometer to observe strong-motion signals and monitor seismic activities in real time. The system covers an offshore area of 50 × 100 km^2^ and has a mean distance of ~14 km between stations, which is comparable to land station networks.

Previous simulation studies^11^,^12^,^13^ of this area have detected amplification of long-period motions and propagation of these motions to the populated land areas. These studies have suggested that the amplification may have been caused by an accretionary prism composed of sedimentary layers with low seismic velocity^14^ on the continental slope from the trench axis to the coastline. Propagation of long-period motions near subduction zones has also been simulated from southwestern Canada to northern California^15^, off Guerrero–Oaxaca in southern Mexico^16^, and near Hokkaido in northeastern Japan^17^. Other recent simulation studies in eastern and southeastern Japan^18^,^19^ reported development of long-period later phases due to the oceanic layer and sediments. However, relatively few observational studies of long-period motions in ocean-bottom areas have been conducted. Boore^20^ demonstrated, using ocean-bottom data, that surface waves composed of late-arrival and long-period motions were converted from body waves at the edge of the Los Angeles basin. As other observational studies, we investigated long-period motions in the Nankai trough area for a terrestrial landslide source by using ocean-bottom data^21^ to distinguish the features of seismic wavefields from those of natural earthquakes and to detect signals from future submarine landslides at ocean-bottom stations. The results showed amplified long-period motions due to seawater and sediment layers at ocean-bottom stations. However, the results did not provide clear evidence on amplification of observation data because only two land station datasets at the rock site were used. Moreover, the results showed prolonged long-period motions of more than 60 s at near source land stations, which is significantly longer than the motions recorded from local seismic events, since the source time function of landslides had a long duration. Thus, the results of that study could not provide a comparison of the prolongation at ocean-bottom stations with those at land stations, nor did they show the process of prolongation during the propagation at the landslide source.

Direct observations and analyses in ocean-bottom areas would contribute to understanding the generation and development of long-period motions and quantitatively evaluating their effects on land areas. Evaluating long-period motions may also contribute to improving the source analyses of large earthquakes that radiate long-period seismic waves. We report here observations of distinct long-period ocean-bottom motions with large amplitudes and long durations in the Nankai trough during a moderate inland event (*M*w 5.8) in 2013. This was the largest-magnitude event near the Nankai trough since the initiation of ocean-bottom observations in 2010. Strong-motion data for an inland earthquake provides a better opportunity for comparing motions on land with those in ocean-bottom areas than does a suboceanic earthquake because motions from a suboceanic earthquake are developed in ocean areas near the source and propagate to land areas, which makes it difficult to observe differences in the motions between land and ocean areas. In this study, we conducted numerical simulations of characteristic features of seismic waveforms at the ocean-bottom stations in the long-period band and evaluated the effects of oceanic sediment layers on the seismic wavefields. We primarily focus on the long-period band of 10–20 s because the simulation can reproduce observations in periods of more than 10 s and ocean-bottom motions show significant amplification and a high S/N ratio in periods of less than 20 s. This long-period band is important for discussing seismic wavefields in deep ocean areas because waveforms can be affected by a seawater layer^22^. This period band is also important for magnitude estimations by using long-period waveform amplitudes such as surface-wave magnitude (*M*s) which is generally measured utilising the amplitudes of Rayleigh waves with a period of 20 s.

## Long-period motions observed in deep ocean-bottom areas

**Figure 1:**
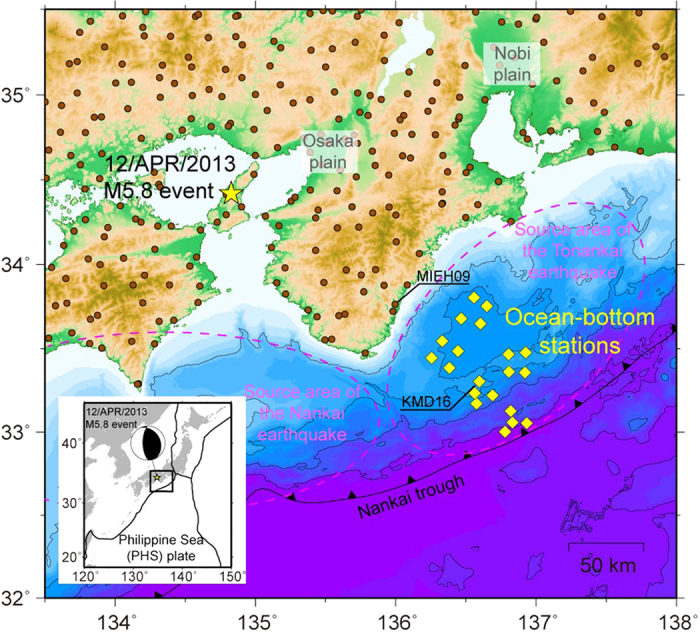
Location map. The yellow star indicates the epicentre of the 2013 inland event (*M*w 5.8). The yellow diamonds and brown circles indicate the locations of ocean-bottom and land stations, respectively. The black contour lines indicate the seafloor topography at intervals of 1000 m. The source areas of the Tonankai and Nankai subduction earthquakes are indicated by dashed purple lines. The frontal line indicates the Nankai trough where the Philippine Sea plate is subducting beneath the Amur plate. The source mechanism is shown with the tectonic plates around the Japanese Islands in the bottom left inset. The map, including the inset, was created using the software Generic Mapping Tools^46^.

Land and ocean-bottom seismic networks in southwestern Japan recorded strong-motion accelerations of several tens to >500 Gal during a moderate earthquake (*M*w 5.8, depth 11 km) on 12 April 2013 (Fig. 1). The earthquake was considered an aftershock of the devastating 1995 Kobe earthquake (*M*w 6.8), as its epicentre was located near the source fault of that earthquake. The observed waveforms at the ocean-bottom stations, located 170 km southeast of the source, were significantly different from those at the land stations in terms of amplitudes and coda waveforms.

**Figure 2:**
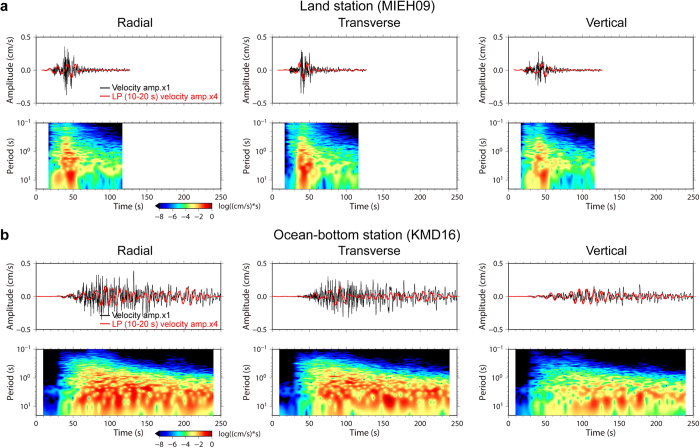
Observed velocity waveforms and spectrograms at land and ocean-bottom strong-motion stations. (**a**) Observation results for land station MIEH09. The upper and lower panels present velocity waveforms and their spectrograms for the 2013 event. The black traces in the upper panels are velocity waveforms obtained from integration of the accelerogram after applying a noncausal six-order high-pass filter with a corner period of 30 s to suppress long-period noise. The red traces indicate velocity waveforms in the long-period band of 10–20 s. (**b**) Observation results for ocean-bottom station KMD16.

Figure 2 shows typical examples of velocity waveforms in the period band of <30 s at land surface station MIEH09 (Fig. 1), located near the coastline, and at station KMD16, located in the centre of the ocean-bottom array at a water depth of 2,000 m. The original land and ocean-bottom data were 100-Hz event-trigger and 200-Hz continuous data, respectively. The dominant period of velocities at MIEH09 and KMD16 was around 7 s and 5 s, respectively.

The waveforms at MIEH09 had a simpler shape with a short coda, indicating more rapid decay of seismic amplitudes after the peak amplitude of the main phase, compared to those at KMD16. The spectrogram for station MIEH09 exhibited high seismic energy, with a short duration of at most 30 s (Fig. 2a), while KMD16 exhibited a much longer duration of >100 s (Fig. 2b). The filtered waveforms (red lines, Fig. 2) and spectrograms for the long-period band of 10–20 s exhibit more noticeable differences in the prolongation of coda parts between the land and ocean-bottom stations. High seismic amplitudes and energy levels were not observed in the coda after 60 s in the long-period band in the waveform or the spectrogram for the land station. The horizontal peak amplitude of 0.04 cm/s in the long-period waveforms at KMD16 was nearly the same as that at MIEH09, despite the greater distance of KMD16 from the source. These observations indicate that long-period motions of 10–20 s were amplified and prolonged at the ocean-bottom stations in comparison to those at the land stations.

**Figure 3:**
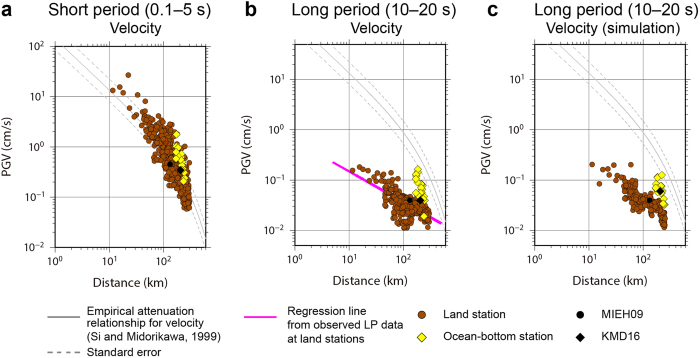
Estimated peak ground velocity (PGV) versus hypocentral distances. Yellow diamonds and brown circles indicate PGVs at ocean-bottom and land stations, respectively, as a function of hypocentral distance from the source to stations assuming a point source approximation. Black diamonds and circles indicate PGVs at KMD16 and MIEH09, respectively. An empirical attenuation relationship and standard deviation for the velocity component for the period band of 0.1–5 s at a stiff soil site^23^ as a function of equivalent hypocentral distance are shown by solid and dashed grey lines, respectively. (**a**) Observed short-period PGV (0.1–5 s). (**b**) Observed long-period PGV (10–20 s). The purple line indicates the regression line obtained for long-period PGV at land stations. (**c**) Simulated long-period PGV (10–20 s).

Figure 3 illustrates the attenuation of peak ground velocity (PGV), determined from the maximum amplitude in the horizontal velocity waveforms as a function of the hypocentral distance from the source to the station assuming a point source approximation. The PGV in the short-period band of 0.1–5 s was similar at the land and ocean-bottom stations (Fig. 3a). The PGV at both the land and ocean-bottom stations agreed approximately with that predicted by empirical equations^23^ for stiff soil in the short-period band as a function of the equivalent hypocentral distance. Differences in amplification between the land and ocean-bottom stations gradually appeared in the long-period band of >3 s, based on analysis of the PGV in various central periods (Figure S1).

Figure 3b shows the PGV in the long-period band of 10–20 s. A regression analysis of log-transformed data for the land stations yielded a regression coefficient of −0.61, which was nearly consistent with the attenuation of surface wave amplitudes in proportion to the reciprocal of the square root of the distance. However, the PGV at the ocean-bottom stations (Fig. 3b) was unexpectedly larger than at the land stations, indicating that the long-period component was significantly amplified in the ocean-bottom areas. At one ocean-bottom station, we observed a PGV five times greater than that at the land station MIEH09. We also found a significant variation in PGVs over 0.02–0.17 cm/s for the ocean-bottom stations. The PGVs at the stations off the trough axis were larger, while those at stations near the trough axis were smaller.

**Figure 4:**
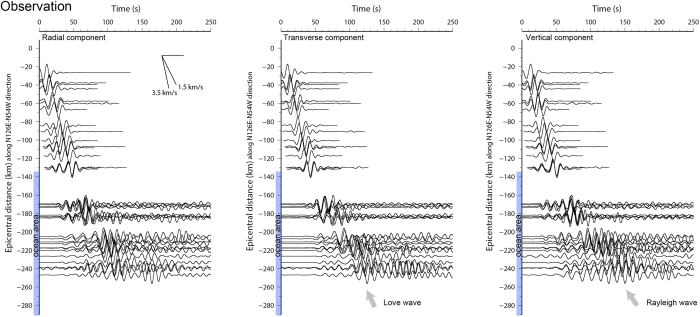
Observed long-period velocity waveforms versus epicentral distances. Black traces represent observed velocity waveforms for the radial (left), transverse (middle), and vertical (right) components in the period band of 10–20 s at land and ocean-bottom stations, in order of epicentral distance. A noncausal six-order band-pass filter was applied to obtain the waveforms. Blue bars in the vertical axis indicate ocean areas.

Figure 4 shows the observed velocity waveforms in the long-period band of 10–20 s at the land and ocean-bottom stations, sorted in order of epicentral distance. Land stations with an epicentral distance of >20 km and a field range of ± 20 km from the epicentre in the direction perpendicular to N126°E–N54°W (dashed red line, Figure S2a) were selected. The propagation speed of the main phases with large amplitudes was ~3.5 km/s on land. However, slow propagation and late arrival of the phases were observed at the ocean-bottom stations. Based on travel time and orbit analyses, the slow propagation phases found in the vertical and transverse components at the ocean-bottom stations were mainly Rayleigh and Love waves, respectively (arrows in Fig. 4). Figure S3 shows the dispersion curves for the fundamental mode of the Rayleigh and Love waves, estimated based on the subsurface structures beneath stations MIEH09 and KMD16. The dispersion curves showed slower group velocities for periods of <15 s for Rayleigh waves and periods of <24 s for Love waves at the ocean-bottom station KMD16 than were estimated at the land station MIEH09. In the period band of <11 s, the dispersion curves for KMD16 indicate that wave packets of Rayleigh and Love waves propagated at a slower group velocity than that of acoustic waves (1.5 km/s). This is probably because sediment layers with low S-wave velocities with thicknesses sensitive to the periods are present in the ocean areas. In addition, for Rayleigh waves, the group velocity decreases with increasing thickness of the water column, as reported in previous theoretical and observational studies^22^,^24^,^25^. The slow propagation of surface waves in ocean areas probably contributes to prolongation of long-period motions at ocean-bottom stations.

## Simulation of long-period motions

We verified significant amplified and prolonged long-period motions in ocean areas for the 2013 earthquake through finite-difference simulations of seismic wave propagation. Previous simulation studies of seismic waves propagating through the Nankai trough have focused on reproducing land station data^11^,^12^,^13^. We reproduced waveforms at ocean-bottom stations by incorporating seawater layer, three-dimensional (3-D) topography of the seafloor, and fluid–solid boundary conditions into our simulations.

Figure S4 shows simulated velocity waveforms of three components in the long-period band of 10–20 s. The stations of the waveforms are located at grid points of the land surface and ocean bottom from the epicentre to N126°E–N54°W (dashed red line, Figure S2a). The simulated waveforms for the land and ocean areas reproduced observed features of amplification and prolongation (Fig. 4). The waveforms at the ocean-bottom stations showed prolonged motions with long durations of >100 s, whereas those at land stations showed simple motions with short durations of at most 30 s. Attenuation of PGVs in the simulated waveforms is shown in Fig. 3c. The PGVs at the land stations were attenuated in proportion to the reciprocal of the square root of the distance, whereas the PGVs at most of the ocean-bottom stations were larger than those at the land stations, which is consistent with the observations (Fig. 3b). The results of several additional simulations using various source mechanisms including strike-slip, dip-slip, the same mechanism as that of the 2013 earthquake except for the strike direction, and the same mechanism as the 1995 Kobe earthquake indicated that the amplification occurring at the ocean-bottom stations could not be explained by source effects, as shown in Figure S5. However, in our forward simulation, we did not obtain a good reproduction of waveforms in the period band of <10 s at the ocean-bottom stations (Figures S6 and S7). Since the precision of our simulation was 2 s for grid dispersion, the waveform differences in the period band may indicate the discrepancies between the present structure model used and actual conditions, implying the need for improving the model.

**Figure 5:**
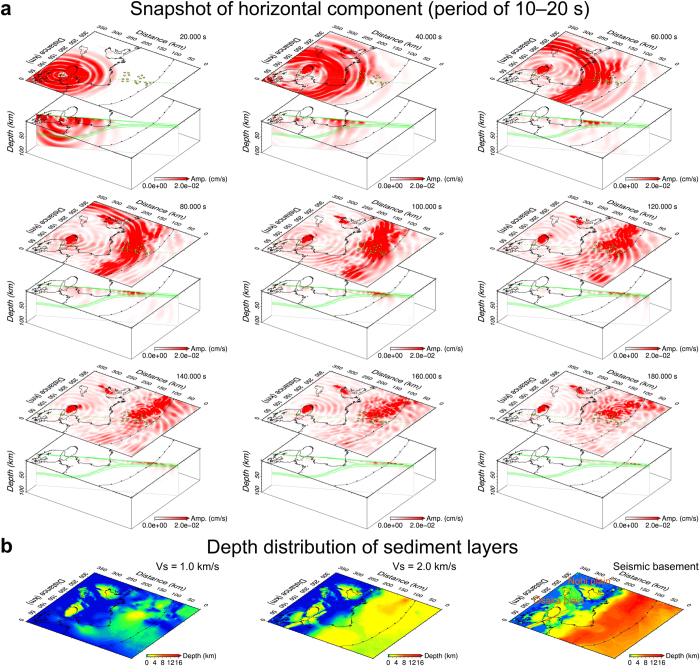
Snapshots from the seismic wave propagation and sediment layers model. (**a**) Distribution of amplitudes (red colour) of the horizontal component in the period band of 10–20 s at elapsed times of 20–180 s. The dashed green lines indicate the surface areas of the cross sections. The green lines in the cross sections show the land and sea surfaces, the seafloor, and the structural boundaries of the seismic basement, oceanic crust (layers 2 and 3) and mantle. The map was created using the software Generic Mapping Tools^46^. (**b**) Depth distribution of the sediment layers for *V*s = 1.0 and 2.0 km/s and the seismic basement from the JSHIS model^39^. The map was created using the software Generic Mapping Tools^46^.

Seismic wave propagation of the horizontal component (the square root of the sum of the squares of the two horizontal components) in the long-period band of 10–20 s is shown in Figure 5 and Supplementary Movie 1. Amplified motions were observed in large basin areas around the Osaka and Nobi plains (Figs 1 and 5b) and also in ocean areas, particularly in the eastern ocean-bottom array. The amplified areas in the ocean were considerably larger than those on land and corresponded to areas with thick sediment layers (Fig. 5b), which confirms the influence of low-velocity sediment layers on seismic amplifications. The snapshots for 60–120 s show amplifications in thick sediment layers after the high-amplitude seismic waves travelled from the land into the ocean. We suggest that the amplified long-period waves are surface waves developed in sediment layers of ocean areas, probably through the same process as in terrestrial sedimentary basins^26^. The thickness of sediment layers in the ocean floor, from the shallow soft layer near the seafloor to the seismic basement (*V*s = 2.7 km/s), varies substantially along the direction of subduction between the coastal area and the trench axis (Figs 5b and S2c). Thus, significant differences in amplifications at the ocean-bottom stations (Fig. 3b,c) are probably due to both attenuation with distance and the thickness of the sediment layers. These amplified long-period motions in ocean areas associated with sediment layers cannot be reproduced by simulation by using a 1-D velocity model. Figure S8 shows synthetic waveforms obtained through the discrete wavenumber method^27^ by using a 1-D model used by the National Research Institute for Earth Science and Disaster Prevention (NIED) to estimate the source mechanism. Although synthetic waveforms produced by the 1-D model can explain those produced by the 3-D model at land stations, they differed significantly at ocean-bottom stations, implying the effect of thickness variation of the sediment layers on waveforms at ocean-bottom stations.

The snapshots also show slower propagation of surface waves in oceans than on land, contributing to prolonged motions at the ocean-bottom stations. Propagation slowed when the seismic wave travelled offshore, where the thickness of the seawater layer and the subsurface sediment layers gradually increases. This change in propagation speed from land to oceans was also verified by travel time analysis of the surface waves. Figure S9 shows the ray paths of the Rayleigh and Love waves and their travel times for periods of 10 and 20 s, calculated from group and phase velocity maps based on an analysis of the dispersion curves (Figure S3) for the land and ocean areas. The distribution of travel times for both the Rayleigh and Love waves for the period of 20 s forms nearly concentric patterns, indicating little difference in the propagation speed between the land and ocean areas. The slightly faster travel times near the trench axis of the Philippine Sea plate are due to high seismic velocities in the oceanic mantle. For the period of 10 s, however, slower travel times were observed in the ocean areas because of the slow phase and group velocities associated with the thick low-velocity sediment layers. For Rayleigh waves, the seawater layer also decreases the velocities. In the finite-difference simulations, we demonstrated this effect using a non-seawater structural model in which the seawater layer of the original model (Figure S2c) was replaced with an air layer (Figure S10a). The difference in wavefields between the non-seawater and seawater models is clearly seen in the simulated waveforms for the vertical component (Figures S4 and S10b). Our results for the effect of seawater correspond to those of recent studies^18^,^19^ on the development of distinct later long-period phases as demonstrated by simulations in eastern and southeastern Japan. For Love waves with a period of 10 s, the slower propagation in the ray analysis was only due to thick low-velocity sediment layers and not a seawater layer, because the seafloor behaves as a free surface for Love wave propagation.

The ray tracings for both Rayleigh and Love waves also identified areas of focusing and defocusing of the rays around the trench axis (Figure S9) caused by lateral variations in phase velocities during propagation. Intense focusing may have amplified seismic motions. In the finite-difference simulations, the distribution of maximum amplitudes in the long-period band for the simulated vertical and transverse components generally had high and low amplitudes in the southwestern ocean areas (Figure S11), possibly explaining the focusing and defocusing areas of Rayleigh and Love wave rays (Figure S9). Near the ocean-bottom stations, the seismic waves were mostly trapped in the thick sediment layers around the stations; the focusing and defocusing areas were not clearly identified there in the distribution of maximum amplitudes.

We also verified amplified and prolonged long-period motions in ocean areas for a simulated offshore event. We assumed that the source was located at the subducting plate interface at a depth of 10 km (Figure S12a and Supplementary Movie 2), near the epicentre of the 1944 Tonankai subduction earthquake. In our simulations, the source had the same seismic moment as that of the 2013 event but had a thrust-type mechanism. The results showed amplified motions for >100 s near ocean-bottom stations (Figure S12a and Supplementary Movie 2). The areas of the amplified and prolonged motions corresponded to the location of thick sediment layers (Figs 5b and S2c). Slow propagation of surface waves was also verified in ocean areas based on travel times for the period of 10 s (Figure S12b).

## Discussion

In the present study, we confirmed amplified and prolonged long-period motions associated with sediment layers at ocean-bottom stations in the period band of 10–20 s. Because thick, low-velocity sediment layers form in subduction areas, these motions may be commonly found in the source area of subduction earthquakes. In this section, we offer a short discussion regarding the possibility that these long-period motions could improperly affect source analyses that use ocean-bottom data for such earthquakes.

The seismic wave propagation in the images in Fig. 5a and the movie file (Supplementary Movie 1) represent a lateral variation of amplification in the ocean areas. In the ocean-bottom stations, the maximum horizontal amplitudes in the period band of 10–20 s from our observation and simulation have variation of 0.02 to 0.16 cm/s (Fig. 3b) and 0.03 to 0.12 cm/s (Fig. 3c), respectively. In the immediate eastern area of the ocean-bottom stations, a significant amplified area was detected in the images and in the movie. The maximum horizontal amplitude in the area was 0.24 cm/s, which is 6 times larger than the observed value at KMD16 ocean-bottom station (Fig. 2) and 10 times larger than that expected from the regression line for the amplitudes of land stations (Fig. 3b). Our simulation results for an offshore event also indicate the same features for the lateral variation at and near the ocean-bottom stations (Figure S12a and Supplementary Movie 2). Recent studies^28^,^29^ have shown that amplification at ocean-bottom stations can result in overestimation of displacement-amplitude magnitudes of earthquakes by ~0.5–0.6, compared with catalogued magnitudes or those estimated from land station data. Because in larger earthquakes, longer-period seismic waves are radiated and are dominantly observed at near-field stations, the amplified long-period motions at ocean-bottom stations in large subduction earthquakes could cause a larger overestimation for magnitude. In addition, the lateral variations of amplifications obtained from our results indicate that long-period motions developed in sediment layers in ocean areas may produce unstable results of magnitude estimations among the stations.

Our simulation results also imply that other challenges may occur in source analysis when evaluating ocean-bottom waveforms, such as in finite fault slip modelling. Because of computational limitations, a layered 1-D velocity structure is often assumed in such analyses for calculating Green’s function. However, the simulated long-period waveforms in ocean areas determined by using the 1-D structure model differed significantly from those determined by using the 3-D model. Figure S13a shows a comparison of synthetic waveforms in the period band of 10–20 s at ocean-bottom stations with an epicentral distance of less than 100 km along the N126°E–N54°W direction. These waveforms were obtained through the finite-difference simulation by using the 3-D model and from the discrete wavenumber method by using the 1-D model with a seawater thickness of 2.6 km and sediment layer thickness of 5.7 km. The same values are used by the Japan Agency for Marine–Earth Science and Technology (JAMSTEC) for routine hypocentre determination of offshore earthquakes. We detected an anomalously slow propagation of seismic waves for the 1-D model that was not found in the waveforms for the 3-D model (Figure S13a). In the period band of 20–30 s, the differences in waveforms between the two models were slight and were found only in the vertical component at land stations (Figure S13b). The significant differences as those found in the period band of 10–20 s were not detected for the 20–30 s band at the ocean-bottom stations, implying that seismic waves around the period of <20 s begin to show sensitivity to differences between 3-D and 1-D models in the ocean areas. These results provide a contrast to those reported in the previous section such that the differences in waveforms between 3-D and 1-D models were not found in the period band of 10–20 s at land stations for the land earthquake (Figure S8). Thus, we emphasise that incorporation of realistic 3-D structures including a seawater layer and sediment layers is more important for simulations in the long-period band of 10–20 s at ocean-bottom stations for subduction earthquake than that at land stations for land earthquakes.

## Conclusion

In this study, we showed observational evidence for long-period motions developed in ocean areas by using deep ocean-bottom strong-motion data for a moderate earthquake (*M*w 5.8). The waveforms and spectrograms demonstrated prolonged and amplified motions that are inconsistent with attenuation patterns of ground motions on land areas. By analysing the PGV, differences in amplification between land and ocean-bottom stations gradually appeared in the period of >3 s. In the period band of 10–20 s, the PGV at the ocean-bottom stations was unexpectedly larger than that at the land stations. Simulated waveforms reproducing observed ocean-bottom data demonstrate substantial contributions of thick, low-velocity sediment layers to development of the amplified and prolonged motions. Ray tracings for the fundamental mode of Rayleigh and Love waves presented slow propagation in ocean areas, which could have contributed to the prolongation of the motions. This development may be common in the source areas of subduction earthquakes where thick, low-velocity sediment layers are present. The long-period motions in ocean areas could improperly affect source analyses such as magnitude estimates and finite fault slip modelling when using ocean-bottom data without careful considerations of the layers to analyse such earthquakes.

## Methods

### Structural model for seismic wave simulations

We used a 3-D structural model for subducting oceanic layers 2 and 3 (i.e., the oceanic crust) and the oceanic mantle by compiling data from reflection and refraction seismic surveys^14^,^30^,^31^,^32^,^33^,^34^,^35^,^36^,^37^ and referring to the hypocenter distributions determined by the Japan Meteorological Agency (JMA). We used the JMA 2001 velocity model^38^ for the structure of the continental crust and mantle. The densities of the oceanic crust and mantle and the continental crust and mantle were determined as a function of *V*p based on empirical relationships^39^. We also used the sediment layer model for *V*p, *V*s, density, and attenuation of P- and S-waves (*Q*p and *Q*s) developed by the Japan Seismic Hazard Information Station (J-SHIS)^40^. In the overlapping region between the J-SHIS model and the oceanic crust model, we gave priority to the latter. The minimum *V*s and *Q*s in the sediment model were 0.35 km/s and 60, respectively. The depth distribution of the upper surface of the seismic basement is shown in Fig. 5b.

For the land and ocean-bottom topography, we used 50- and 500-m mesh data provided by the Geospatial Information Authority of Japan (GSI) and the Japan Oceanographic Data Center (JODC), respectively. We modelled a seawater layer with *Vp* = 1.5 km/s, *Vs* = 0.0 km/s, and *ρ* = 1.0 g/cm^3^. We also modelled an air layer with *Vp* = *Vs* = 0.0 km/s in the structural model to incorporate the land topography. The simulation area and the structural model are shown in Figure S2.

### Seismic-wave simulation methods by using 3-D structural model

We employed the Heterogeneity, Oceanic Layer, and Topography (HOT)-FDM scheme developed by Nakamura *et al.*^41^ to simulate seismic wave propagation in the land and ocean areas. We incorporated 3-D heterogeneities, including seismic wave attenuation, a seawater layer, the seafloor, and the land surface, into the simulation. We also modelled an irregular fluid–solid boundary at the seafloor and land surface in the grid cells^42^. We used F-net solutions (http://www.fnet.bosai.go.jp/top.php) provided by the National Research Institute for Earth Science and Disaster Prevention (NIED) for the source mechanisms. We used a cosine-type pulse with a width of 2.5 s as a source–time function, which is typical for an earthquake of the magnitude of the 2013 earthquake (*M*w 5.8), based on scaling laws. The computational domain covered an area of 355 × 330 km^2^ in and around the source and the stations and extended to a depth of 100 km. The temporal and spatial grid spacings were 0.005 s and 0.1 km, respectively, which is finer than those used in previous studies^11^,^12^,^13^ and in our previous research^21^ in the focused area. The time step was 50,000 steps, corresponding to 250 s. We simulated waveforms with a period of >2 s using a grid spacing of 0.1 km and eight grid points per minimum shear wavelength to suppress grid dispersion due to the discretized structural model. We effectively avoided artificial reflections from the sides and bottom of the computational domain by modelling perfectly matched layers^43^ in our code. We conducted seismic wave simulations with 2,400 processors on the large-scale parallel computational system of the K computer. We generated snapshot data for the long-period band of 10–20 s by applying a non-causal band-pass filter for simulated waveforms at the finite-difference grids (Fig. 5 and S11, 12 and Supplementary Movies 1 and 2).

### Dispersion curves and ray tracing of surface waves

We constructed local 1-D structures at each grid point at horizontal intervals of 1 km from the 3-D structural model of the finite-difference simulations. We calculated dispersion curves for phase and group velocities for the fundamental mode of the Rayleigh and Love waves using the constructed 1-D structures and the program DISPER 80^44^. From the dispersion curves at the grid points, we obtained 2-D phase and group velocity maps of our present study area for each period. After applying a spatial smoothing filter to satisfy the short-wavelength condition, we numerically calculated surface wave equations for the ray paths^45^ using the phase velocity maps and the Runge–Kutta method. We estimated the arrival times of the wave packets from the group velocity maps along the ray paths. At inter-grid points, we interpolated the phase and group velocities and their gradients using those at the surrounding grid points.

## Additional Information

**How to cite this article**: Nakamura, T. *et al.* Long-period ocean-bottom motions in the source areas of large subduction earthquakes. *Sci. Rep.* **5**, 16648; doi: 10.1038/srep16648 (2015).

## Supplementary Material

Supplementary Information

Supplementary Movie 1

Supplementary Movie 2
