# Supplementary material for: Long-period ocean-bottom motions in the source areas of large subduction earthquakes
Source: Sci Rep. 2015 Nov 30;5:16648. doi: 10.1038/srep16648 (PMC4663758; doi:10.1038/srep16648)
Supplement: Supplementary Information [file srep16648-s1.pdf]

Long-period ocean-bottom motions in the source areas of large subduction earthquakes

Takeshi Nakamura[1*], Hiroshi Takenaka[2], Taro Okamoto[3], Michihiro Ohori[4], and Seiji Tsuboi[5]

[1]Research and Development Center for Earthquake and Tsunami, Japan Agency for Marine–Earth Science and Technology, 3173-25 Showa-machi, Kanazawa-ku, Yokohama 236-0001, Japan.
[2]Department of Earth Sciences, Okayama University, 3-1-1 Tsushima-Naka, Kita-ku, Okayama 700-8530, Japan.
[3]Department of Earth and Planetary Sciences, Tokyo Institute of Technology, 2-12-1 Ookayama, Meguro-ku, Tokyo 152-8551, Japan.
[4]Research Institute of Nuclear Engineering, University of Fukui, 1-2-4 Kanawa-cho, Tsuruga City, Fukui 914-0055, Japan.
[5]Center for Earth Information Science and Technology, Japan Agency for Marine–Earth Science and Technology, 3173-25 Showa-machi, Kanazawa-ku, Yokohama 236-0001, Japan.
*e-mail: t_nakamura@jamstec.go.jp

Supplementary movies

Movie 1 (Movie1_2013event.mov): Simulated seismic wave propagation for the 2013 event. The map was created using the software Generic Mapping Tools[1].

Movie 2 (Movie2_offshoreevent.mov): Simulated seismic wave propagation for an offshore event. The map was created using the software Generic Mapping Tools[1].

Supplementary figure legends

Figure S1. Estimated peak ground velocity (PGV) for the central period of 1–4 s versus hypocentral distances. Yellow diamonds and brown circles indicate PGVs at ocean-bottom and land stations, respectively, as a function of hypocentral distance from the source to stations assuming a point source approximation. An empirical attenuation relationship and its standard error for the velocity component in the period band of 0.1–5 s at a stiff soil site[2] as a function of equivalent hypocentral distance are shown by the solid and dashed lines, respectively.

Figure S2. Simulation area and structural model. (a) The red rectangle indicates the simulation area. The A–A′ indicates the line connecting the epicentral location of the source and the ocean-bottom station. The map was created using the software Generic Mapping Tools[1]. (b) Depth distribution for the upper surface of oceanic layer 2 and the mantle. Black contour lines indicate intervals of 10 km. Thick white lines indicate the reflection and refraction survey lines used to construct the structural model of the oceanic layers and mantle. The map was created using the software Generic Mapping Tools[1]. (c) Cross-section of the constructed structural model along the A–A′ line. The colour indicates *P*-wave velocity.

Figure S3. Dispersion curves at land (MIEH09) and ocean-bottom (KMD16) strong-motion stations. The upper and lower panels show curves for the fundamental modes of the Rayleigh and Love waves, respectively. Dashed and solid lines indicate the dispersion curves for the phase and group velocities, respectively.

Figure S4. Simulated long-period velocity waveforms versus epicentral distances. Black traces represent the simulated velocity waveforms for the radial (left), transverse (middle), and vertical (right) components in the period band of 10–20 s at land and ocean-bottom stations, in order of epicentral distance. A noncausal six-order band-pass filter was applied to obtain the waveforms. Blue bars in the vertical axis indicate ocean areas.

Figure S5. Estimated peak ground velocity (PGV) versus hypocentral distances for various source mechanisms. Yellow diamonds and brown circles indicate PGVs at ocean-bottom and land stations from simulations, respectively, as a function of hypocentral distance from the source to stations assuming a point source approximation. An empirical attenuation relationship and standard deviation for the velocity component for the period band of 0.1–5 s at a stiff soil site[2] as a function of equivalent hypocentral distance are shown by solid and dashed grey lines, respectively. Panels from the left to the right show PGV (10–20 s) for the strike-slip mechanism of (strike, dip, rake)=(224°, 76°, 178°), which is the same

as that of the 1995 earthquake estimated by Japan Meteorological Agency (JMA), PGV for the same mechanism as the 2013 earthquake except for the strike direction (269°, 65°, 102°), and PGV for the dip-slip mechanism (179°, 90°, 90°) and strike-slip mechanism (179°, 90°, 180°), which are the same strike direction as that of the 2013 earthquake, respectively.

Figure S6. Observed velocity waveforms versus epicentral distance in the period band of 2–10 s. Black traces represent the observed velocity waveforms for the radial (left), transverse (middle), and vertical (right) components at land and ocean-bottom stations, in order of epicentral distance. Blue bars in the vertical axis indicate ocean areas.

Figure S7. Simulated velocity waveforms versus epicentral distance in the period band of 2–10 s. Black traces represent the simulated velocity waveforms for the radial (left), transverse (middle), and vertical (right) components at land and ocean-bottom stations, in order of epicentral distance. Blue bars in the vertical axis indicate ocean areas.

Figure S8. Comparison of synthetic waveforms produced by the 3-D model with those by the 1-D model for the 2013 earthquake. Blue and red traces represent synthetic waveforms for the period band of 10–20 s obtained from the finite-difference method (FDM) simulation by using the 3-D model and those from the discrete wavenumber method (DWM) simulation[3] by using the 1-D model, respectively. The 1-D model was used by the National Research Institute for Earth Science and Disaster Prevention (NIED) to estimate the source mechanism of the 2013 earthquake. Left, middle, and right panels show the radial, transverse, and vertical components at stations along the N126°E–N54°W direction, respectively, in order of epicentral distance. All stations used for simulation by the 1-D model are located in the land surface because no seawater is present in the model. Blue bars in the vertical axis indicate ocean areas.

Figure S9. Ray paths and travel times of Rayleigh and Love waves. The upper and lower panels show the ray paths and travel times of the fundamental modes of the Rayleigh and Love waves, respectively. The ray paths were calculated for each degree from the source for periods of 10 and 20 s. The travel times were calculated from group velocities along the ray path. The map was created using the software Generic Mapping Tools[1].

Figure S10. Simulation results using a non-seawater structural model. (a) Structural model that did not include a seawater layer, replacing the water layer of the original structural model (Figure S2c) with an air layer. Colour indicates P-wave velocity. (b) Vertical velocity waveforms simulated using the non-seawater structural model. A noncausal six-order band-pass filter in the period range of 10–20 s was applied to obtain the waveforms. (c) Ray

paths and travel times of Rayleigh waves with periods of 10 and 20 s using the non-seawater model. The map was created using the software Generic Mapping Tools[1].

Figure S11. Maximum amplitudes of simulated long-period velocity waveforms. The left and right panels show the maximum amplitude distribution in the period band of 10–20 s for transverse and vertical components, respectively, for all time steps for the 2013 event. The amplitude which is normalized based on the regression analysis for observed PGV on land (Figure 3) is plotted in the map view at the ground surface and seafloor and in the cross-section. Blue and yellow circles show ocean areas with large and small maximum amplitudes, respectively, corresponding to focusing and defocusing areas of rays from the source (Figure S9). The map was created using the software Generic Mapping Tools[1].

Figure S12. Simulation results for an offshore event. (a) Horizontal component for the period band of 10–20 s at elapsed times of 20–180 s for an offshore event. The source was located near the epicentre of the 1944 Tonankai earthquake. Amplitudes are indicated in red colour. Green lines in the cross section show the land and sea surfaces, the seafloor, and the structural boundary of the seismic basement, oceanic crust (layers 2 and 3), and mantle. The map was created using the software Generic Mapping Tools[1]. (b) Ray paths and travel times of Rayleigh and Love waves for periods of 10 and 20 s. The map was created using the software Generic Mapping Tools[1].

Figure S13. Comparison of synthetic waveforms produced by using the 3-D structural model with those by the 1-D model for an offshore event. Blue and green traces represent synthetic waveforms obtained from the finite-difference method (FDM) simulation by using the 3-D model and those from the discrete wavenumber method (DWM) simulation[3] by using the 1-D model with seawater and sediment layers used by the Japan Agency for Marine–Earth Science and Technology (JAMSTEC) for routine hypocentre determination of offshore earthquakes, respectively. Left, middle, and right panels show the radial, transverse, and vertical components at stations with an epicentral distance of less than 100 km along the N126°E–N54°W direction, respectively. All stations for simulation by the 1-D model are located at the ocean bottom because of no land surface is present in the model. Blue bars in the vertical axis indicate ocean areas. (a) Synthetic waveforms for the period band of 10–20 s. (b) Synthetic waveforms for the period band of 20–30 s.

Supplementary References

1. Wessel, P. & Smith, W. H. F. New, improved version of Generic Mapping Tools released, *Eos Trans. Am. Geophys. Union* **79**, 579 (1998).
2. Si, H. & Midorikawa, S. New attenuation relations for peak ground acceleration and velocity considering effects of fault type and site condition. *J. Struct. Construct. Eng.* **523**, 63–70 (1999).
3. Nakamura, T. & Takenaka, H. A numerical analysis of seismic waves for an anisotropic fault zone. *Earth Planets Space* **58**, 569–582 (2006).

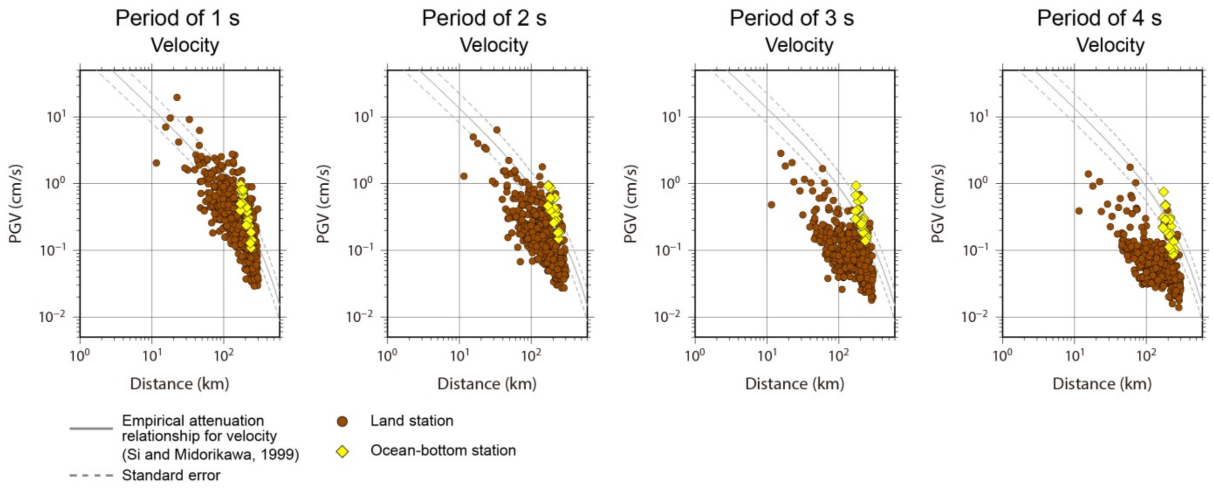
Period of 1 s
Velocity
Period of 2 s
Velocity
Period of 3 s
Velocity
Period of 4 s
Velocity
PGV (cm/s)
Distance (km)
Empirical attenuation relationship for velocity (Si and Midorikawa, 1999)
Standard error
Land station
Ocean-bottom station

Figure S1

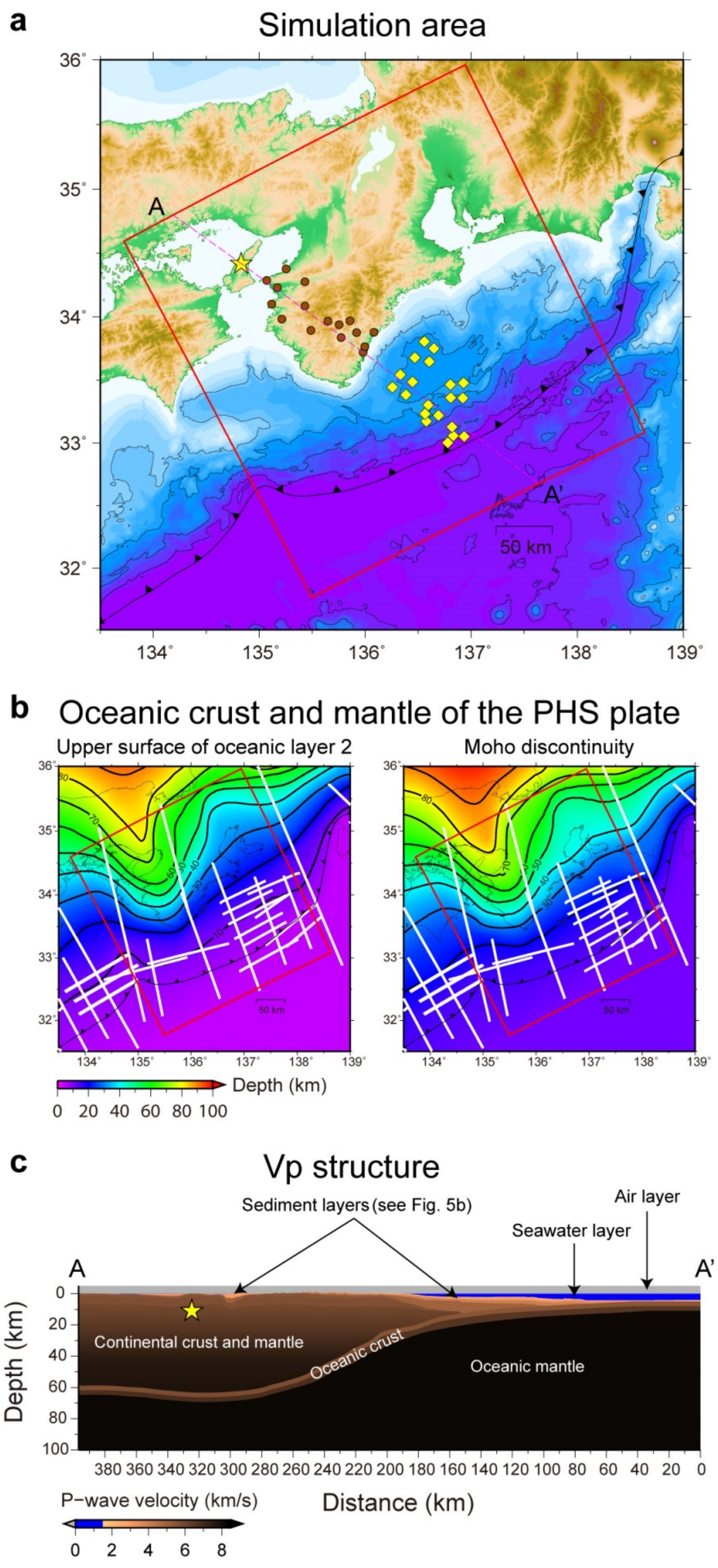
a
Simulation area
A
A'
50 km
b
Oceanic crust and mantle of the PHS plate
Upper surface of oceanic layer 2
Moho discontinuity
Depth (km)
0 20 40 60 80 100
c
Vp structure
Sediment layers (see Fig. 5b)
Air layer
Seawater layer
A
A'
Continental crust and mantle
Oceanic crust
Oceanic mantle
Depth (km)
Distance (km)
P−wave velocity (km/s)
0 2 4 6 8

Figure S2

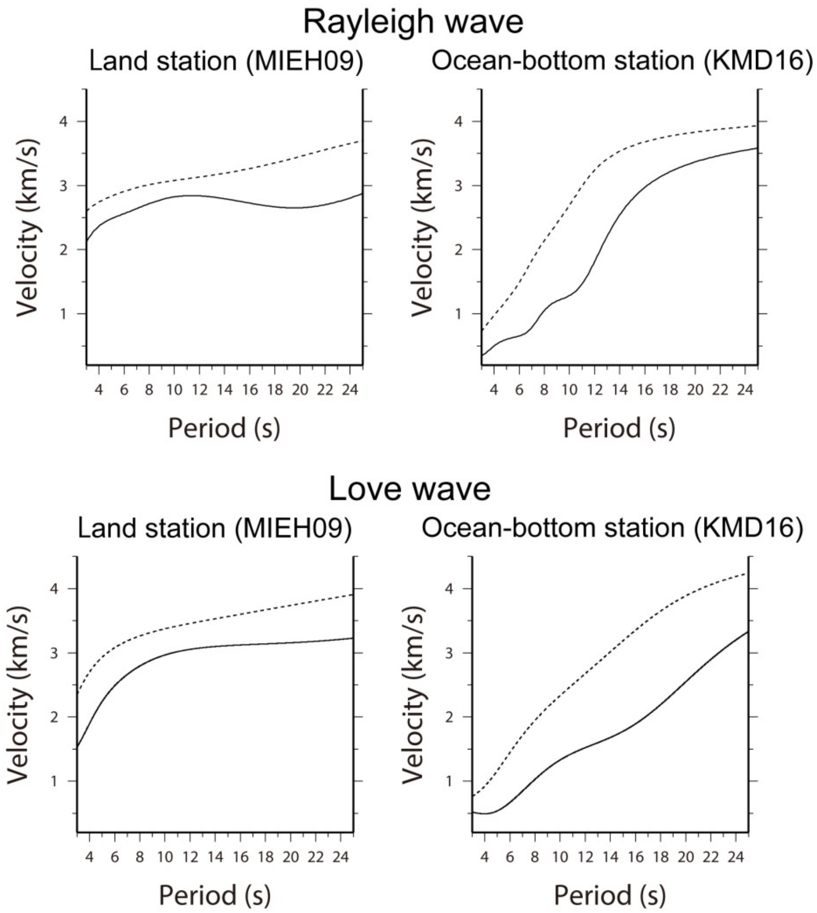
Rayleigh wave
Land station (MIEH09)
Ocean-bottom station (KMD16)
Velocity (km/s)
Period (s)
Love wave
Land station (MIEH09)
Ocean-bottom station (KMD16)
Velocity (km/s)
Period (s)

Figure S3

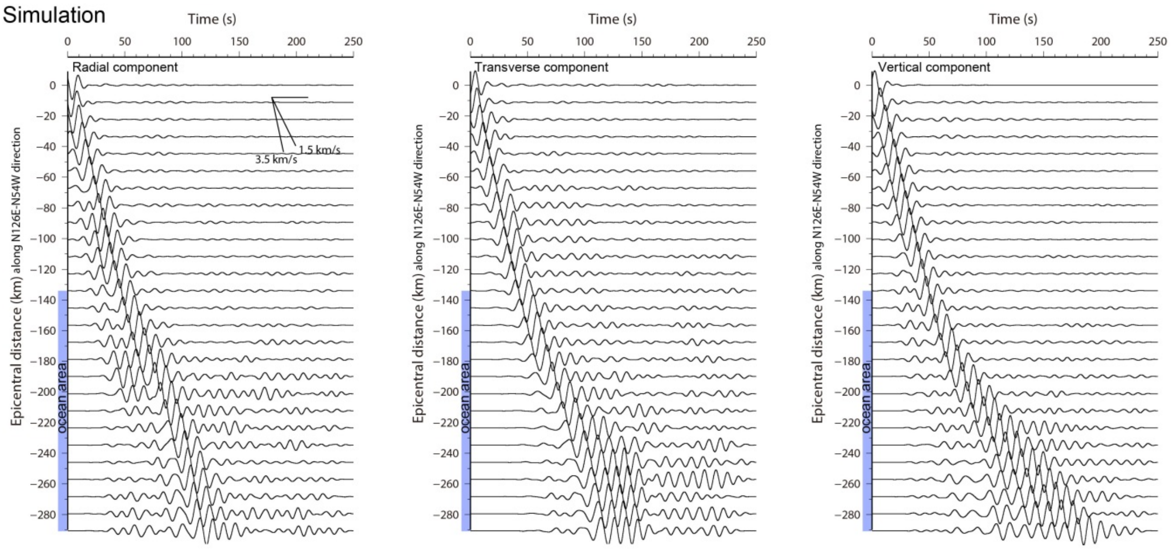

Figure S4

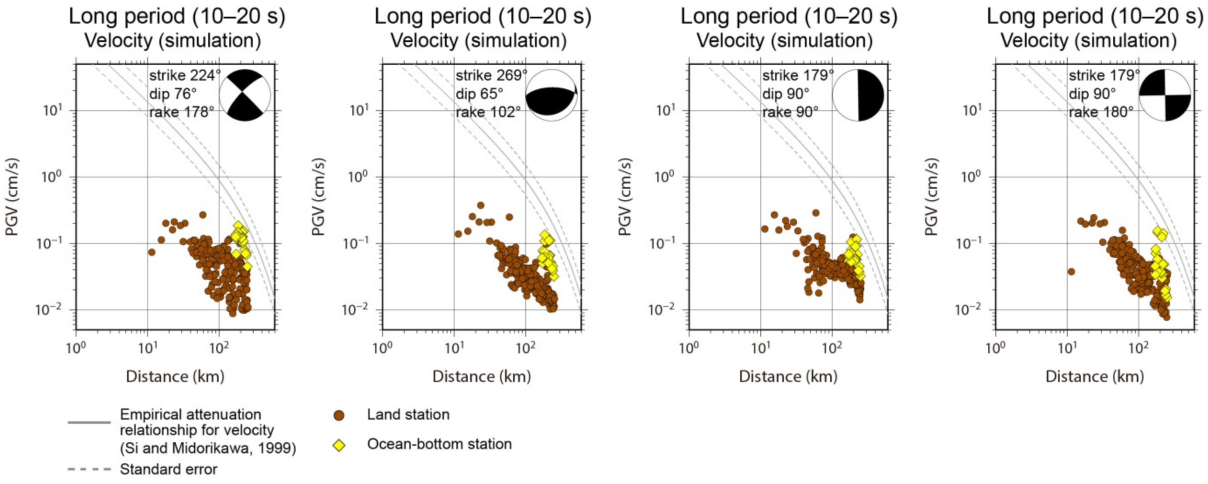

Figure S5

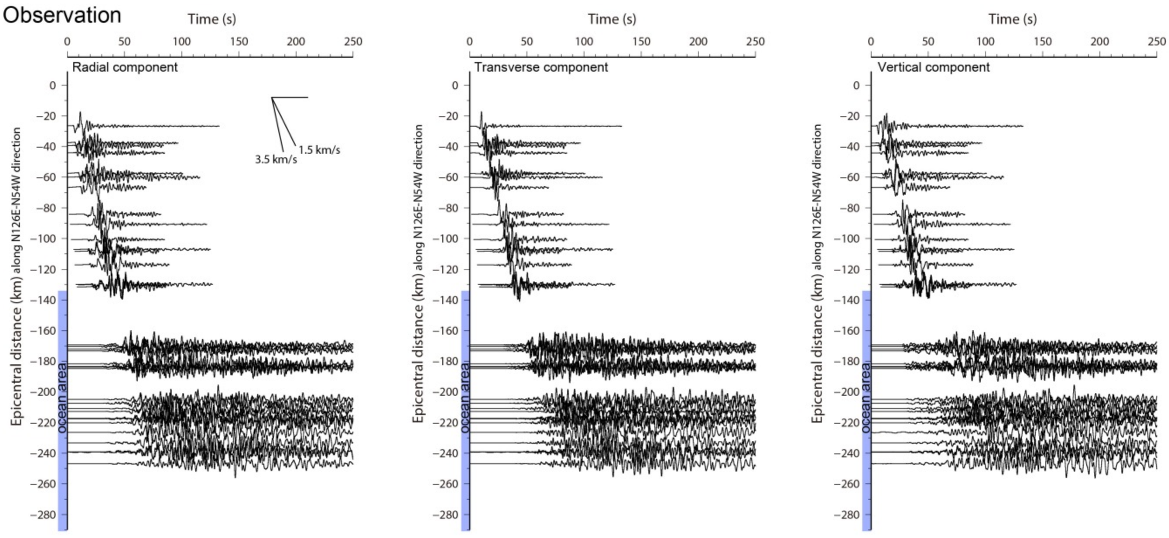

Figure S6

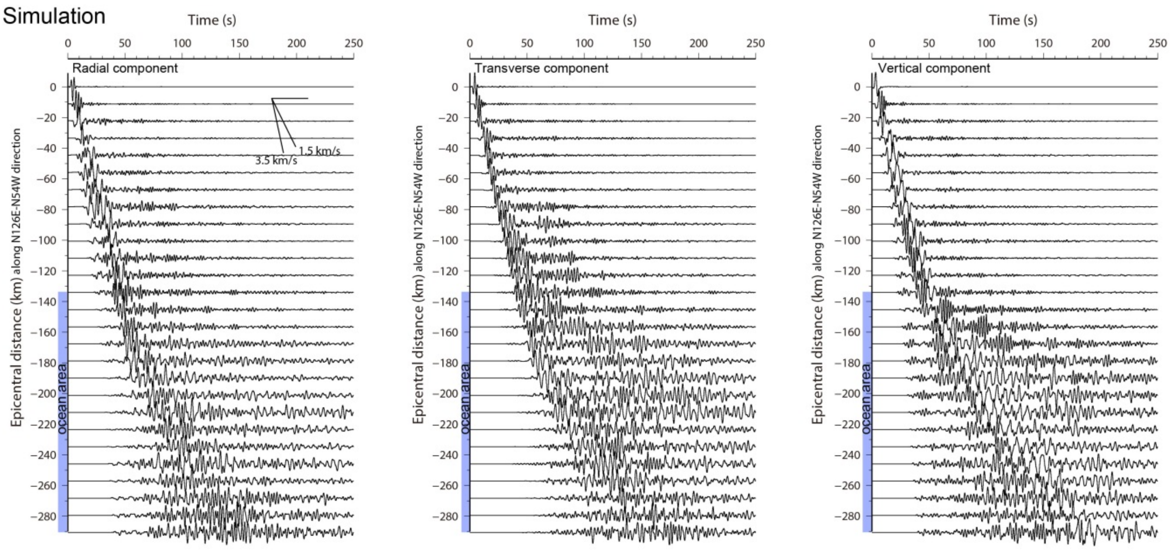

Figure S7

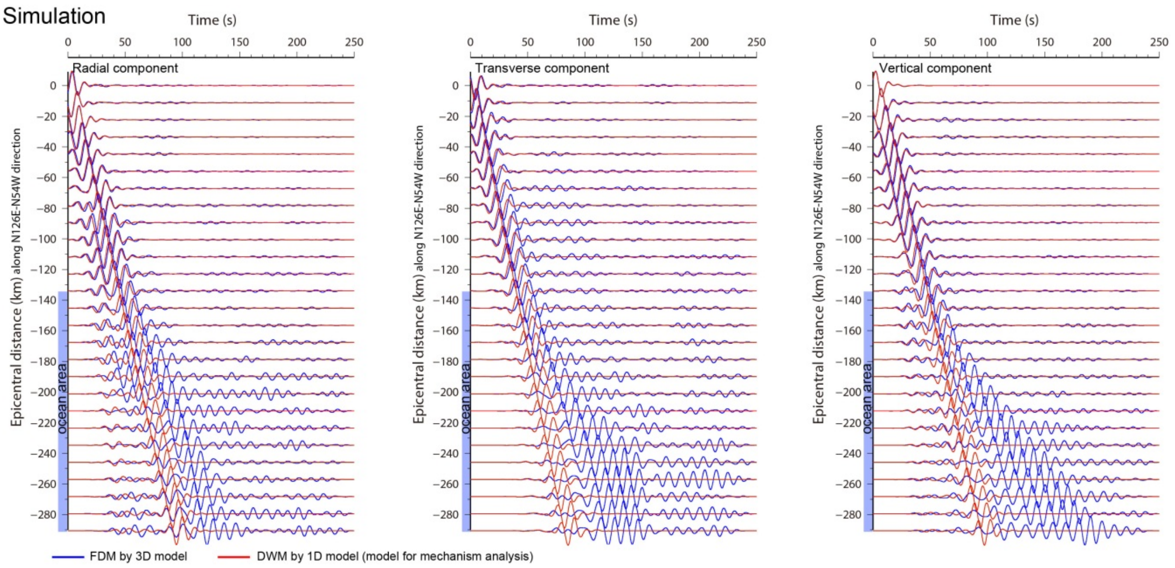

Figure S8

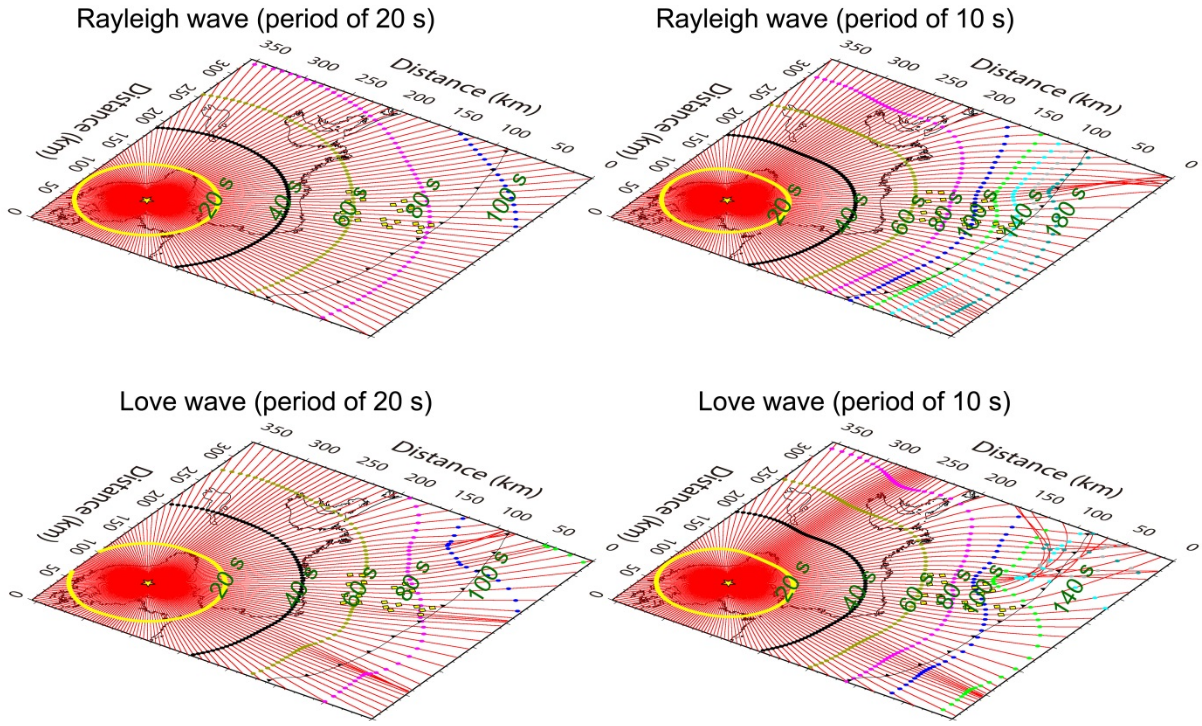

Figure S9

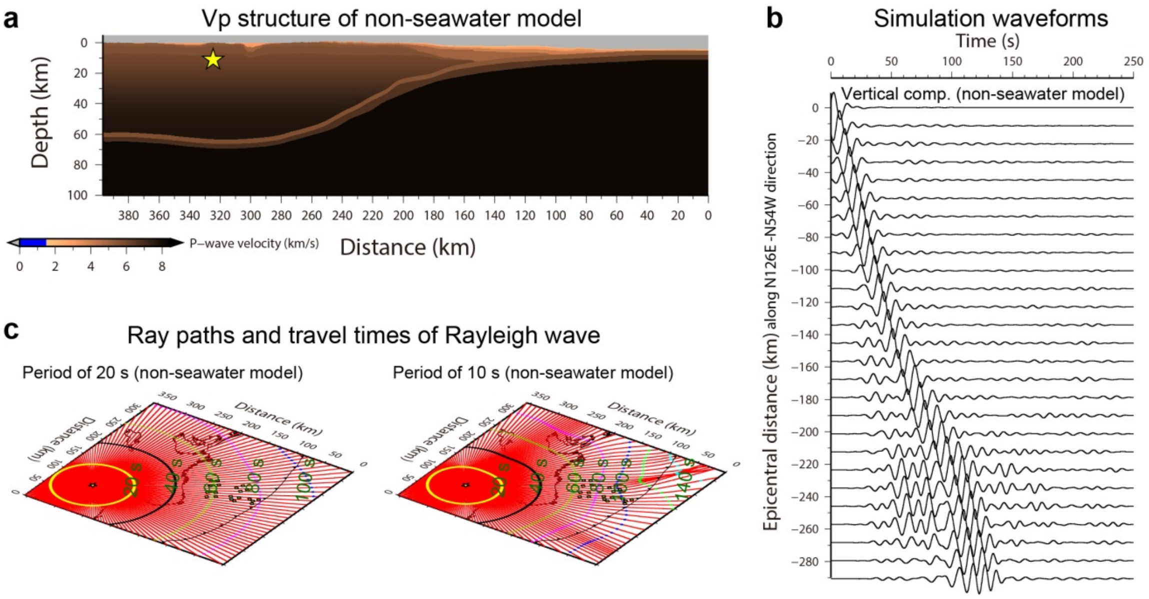

Figure S10

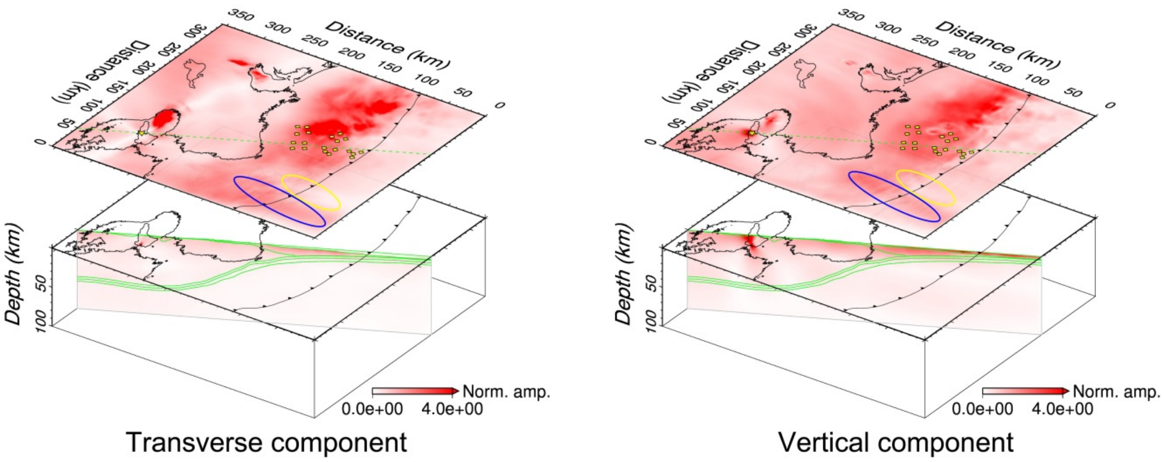

Figure S11

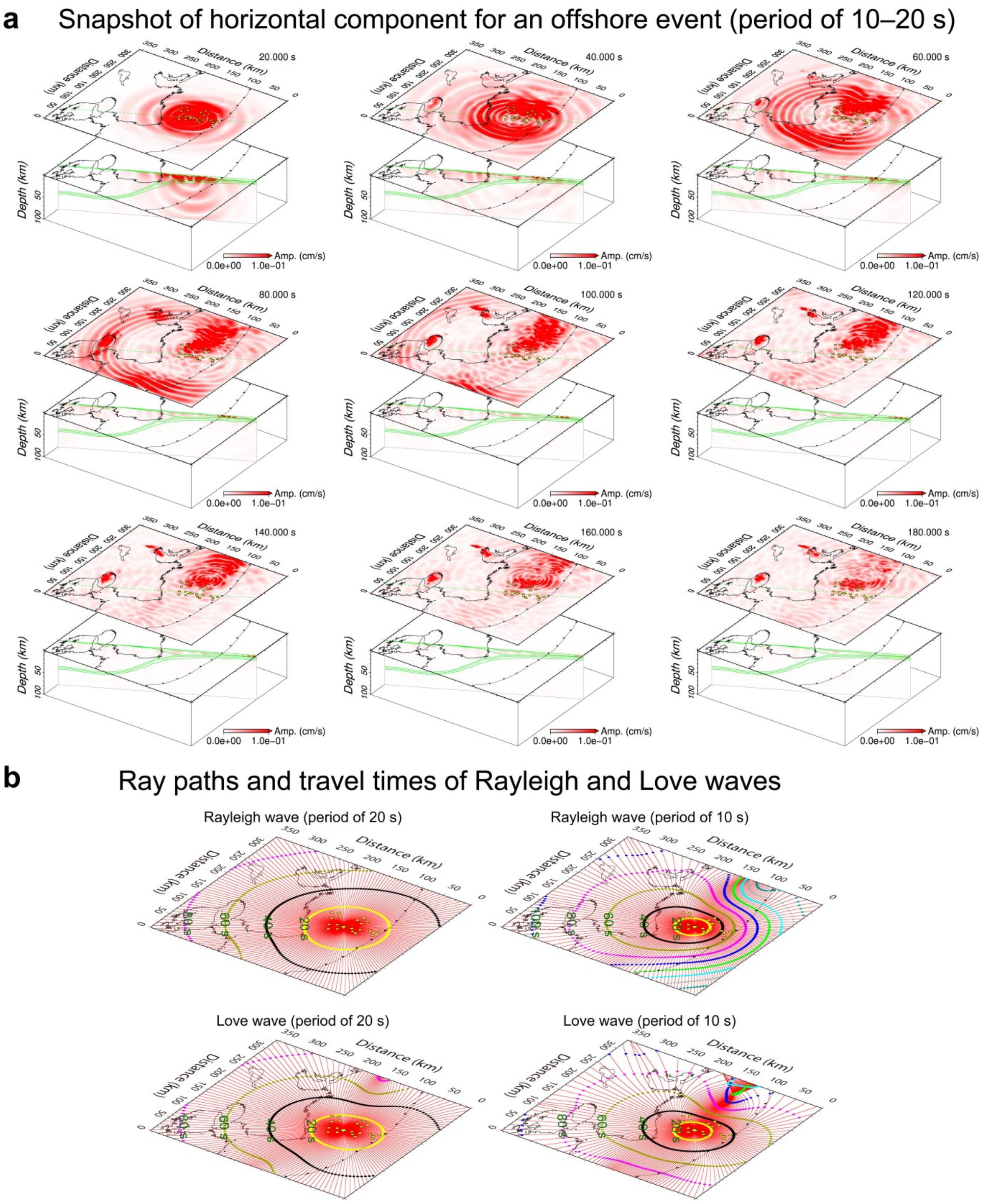
a
Snapshot of horizontal component for an offshore event (period of 10–20 s)
20.000 s
40.000 s
60.000 s
80.000 s
100.000 s
120.000 s
140.000 s
160.000 s
180.000 s
Distance (km)
Depth (km)
Amp. (cm/s)
0.0e+00
1.0e–01
b
Ray paths and travel times of Rayleigh and Love waves
Rayleigh wave (period of 20 s)
Rayleigh wave (period of 10 s)
Love wave (period of 20 s)
Love wave (period of 10 s)

Figure S12

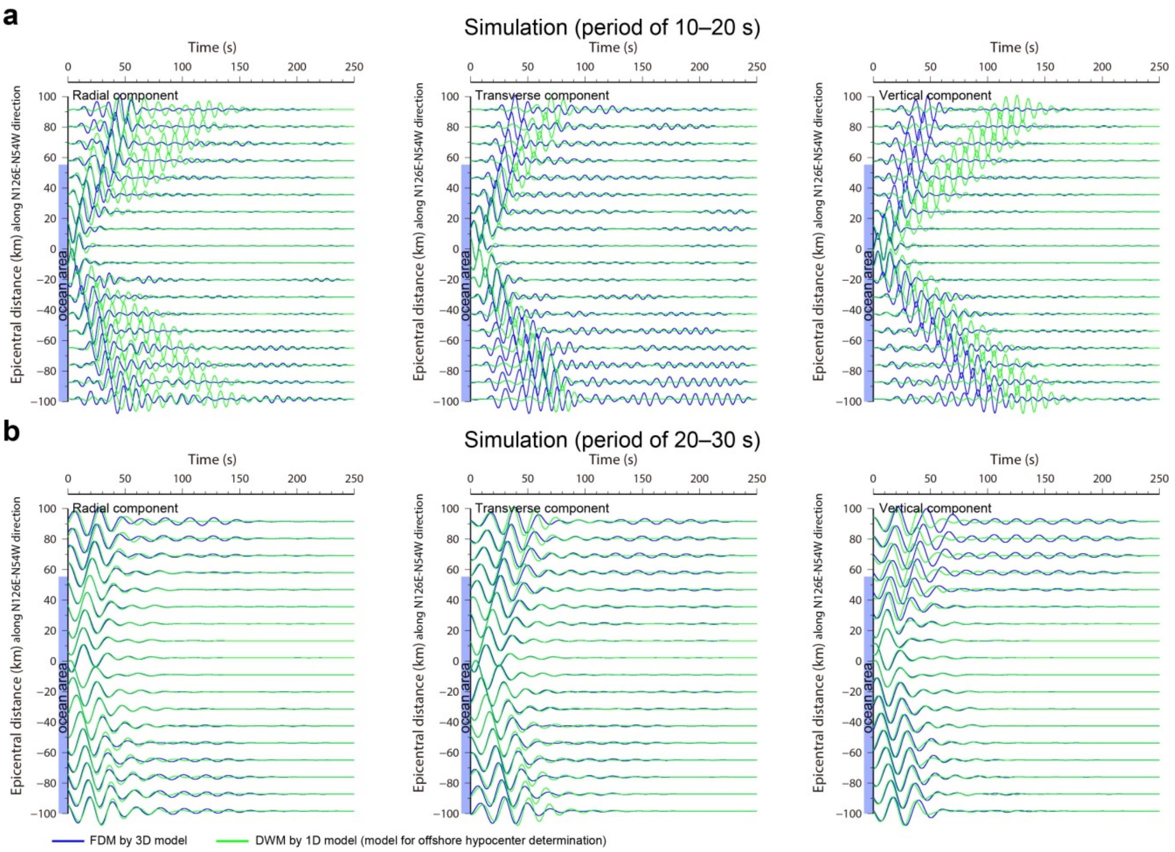
a
Simulation (period of 10–20 s)
b
Simulation (period of 20–30 s)
Time (s)
Radial component
Transverse component
Vertical component
Epicentral distance (km) along N126E-N54W direction
ocean area
FDM by 3D model
DWM by 1D model (model for offshore hypocenter determination)

Figure S13
